# Supplementary material for: FastqPuri: high-performance preprocessing of RNA-seq data
Source: BMC Bioinformatics. 2019 May 3;20:226. doi: 10.1186/s12859-019-2799-0 (PMC6500068; doi:10.1186/s12859-019-2799-0)
Supplement: Supplementary file 2 — Archive of FastqPuri. Archive containing all files needed to install and run FastqPuri v1.0.6. Date stamp March 22, 2019. (GZ 47,819 kb) [file 12859_2019_2799_MOESM2_ESM.gz › FastqPuri-1.0.6/html/trim_8c.html]

FastqPuri: src/trim.c File Reference


FastqPuri


- src

Macros |
Functions |
Variables

trim.c File Reference

trims/filter sequences after Quality, N's contaminations.
More...

`#include <string.h>`  
`#include <stdlib.h>`  
`#include "trim.h"`  
`#include "str_manip.h"`  
`#include "defines.h"`  
`#include "config.h"`  
`#include "struct_trimFilter.h"`

Include dependency graph for trim.c:

|  |  |
| --- | --- |
| Macros | |
| #define | TRIM\_STRING   20 |
|  | |

|  |  |
| --- | --- |
| Functions | |
| static int | no\_N (Fq\_read \*seq) |
|  | checks if a sequence contains any non standard base callings (N's) More... |
|  | |
| static int | Nfree\_Lmer (Fq\_read \*seq, int minL) |
|  | Finds the largest Nfree sub-seq and keeps it if larger than minL. More... |
|  | |
| static int | Ntrim\_ends (Fq\_read \*seq, int minL) |
|  | trims a read if N's are at the ends and the remaining sub-seq >= minL More... |
|  | |
| static int | no\_lowQ (Fq\_read \*seq, int minQ) |
|  | checks if a sequence contains lowQ nucleotides More... |
|  | |
| static int | Qtrim\_ends (Fq\_read \*seq, int minQ, int minL) |
|  | trims a read if lowQs are at the ends and remaining sub-seq >= minL More... |
|  | |
| static int | Qtrim\_frac (Fq\_read \*seq, int minQ, int nlowQ) |
|  | accepts the sequence as is if there are less than nlowQ More... |
|  | |
| static int | **Qtrim\_endsfrac** (Fq\_read \*seq, int minQ, int minL, int nlowQ) |
|  | |
| int | Qtrim\_global (Fq\_read \*seq, int left, int right, char type) |
|  | trims left from the left and right from the right More... |
|  | |
| static int | align\_uint32 (Fq\_read \*seq, Ad\_seq \*ptr\_adap, bool all) |
|  | alignment search between a fq read, and an adapter sequence, with a seed of 8 nucleotides. More... |
|  | |
| static int | align\_uint64 (Fq\_read \*seq, Ad\_seq \*ptr\_adap) |
|  | Alignment search between a fq read, and an adapter sequence, w with a seed of 8 nucleotides. More... |
|  | |
| int | trim\_adapter (Fq\_read \*seq, Ad\_seq \*adap\_list) |
|  | trims sequence based on presence of N nucleotides More... |
|  | |
| int | trim\_sequenceN (Fq\_read \*seq) |
|  | trims sequence based on presence of N nucleotides More... |
|  | |
| int | trim\_sequenceQ (Fq\_read \*seq) |
|  | trims sequence based on lowQ base callings More... |
|  | |
| bool | is\_read\_inTree (Tree \*tree\_ptr, Fq\_read \*seq) |
|  | check if Lread is contained in tree. It computes the score for the read and its reverse complement; if one ot them exceeds the user selected threshold, it returns true. Otherwise, it returns false. More... |
|  | |
| bool | is\_read\_inBloom (Bfilter \*ptr\_bf, Fq\_read \*seq, Bfkmer \*ptr\_bfkmer) |
|  | checks if a read is in Bloom filter. It computes the score for the read and returns true if it exceeds the user selected threshold. Returns false othersise. More... |
|  | |

|  |  |
| --- | --- |
| Variables | |
| int | Nencode |
|  | |
| Iparam\_trimFilter | par\_TF |
|  | |

## Detailed Description

trims/filter sequences after Quality, N's contaminations.

Author
:   Paula Perez paula.nosp@m.pere.nosp@m.zrubi.nosp@m.o@gm.nosp@m.ail.c.nosp@m.om

Date
:   24.08.2017

## Macro Definition Documentation

## ◆ TRIM\_STRING

#define TRIM\_STRING   20

maximal length of trimming info string.

## Function Documentation

## ◆ align\_uint32()

|  |  |  |  |
| --- | --- | --- | --- |
| static int align\_uint32 | ( | Fq\_read \* | *seq*, |
|  |  | Ad\_seq \* | *ptr\_adap*, |
|  |  | bool | *all* |
|  | ) |  |  |

static

alignment search between a fq read, and an adapter sequence, with a seed of 8 nucleotides.

This function checks whether there is adapter contamination in a given read. It works stand alone if the adapter is shorter than 16 nucleotides, and is called from align\_uint64 when no 16-nucleotides long seeds are found. The criteria are the same as in align\_uint64, the seed length being 8-nucleotides long instead of 16. See the **align\_uint64** documentation for more details.

Parameters
:   |  |  |
    | --- | --- |
    | seq | pointer to **Fq\_read** |
    | ptr\_adap | pointer to **Ad\_seq** |
    | all | true if the whole read has to be sweeped, false if only the ends. When this function is called from align\_uint64, only the ends need to be considered. |

Returns
:   -1 error, 0 discarded, 1 accepted as is, 2 accepted and trimmed

Note
:   Global input parameters from par\_TF are also used

See also
:   Adapter
:   Iparam\_trimFilter
:   align\_uint64
:   pack\_adapter
:   obtain\_score

## ◆ align\_uint64()

|  |  |  |  |
| --- | --- | --- | --- |
| static int align\_uint64 | ( | Fq\_read \* | *seq*, |
|  |  | Ad\_seq \* | *ptr\_adap* |
|  | ) |  |  |

static

Alignment search between a fq read, and an adapter sequence, w with a seed of 8 nucleotides.

Parameters
:   |  |  |
    | --- | --- |
    | seq | pointer to **Fq\_read** |
    | ptr\_adap | pointer to **Ad\_seq** |

Returns
:   -1 error, 0 discarded, 1 accepted as is, 2 accepted and trimmed

Note
:   Global input parameters from par\_TF are used

See also
:   Adapter
:   Iparam\_trimFilter
:   align\_uint32
:   pack\_adapter
:   obtain\_score

This function checks whether there is adapter contamination in a given read. We start by looking for 16-nucleotides long seeds, where a user defined number of mismatches is allowed. If found, a score is computed. If the score is larger than the user defined threshold and the number of matched nucleotides exceeds MIN\_NMATCHES (12), then the read is trimmed if the remaining part is longer than minL (user defined) and discarded otherwise. If no 16-nucleotides long seeds are found, we proceed with 8-nucleotides long seeds (see **align\_uint32**) and apply the same criteria to trim/discard a read. A list of possible situations follows, to illustrate how it works (minL=25, mismatches=2):

ADAPTER: CAAGCAGAAGACGGCATACGAG

REV\_COM: AGATCGGAAGAGCTCGTATGCC

CASE1A: CACAGTCGATCAGCGAGCAGGCATTCATGCTGAGATCGGAAGAGATCGTATG

||||||||||||X|||----

AGATCGGAAGAGCTCGTATG

- Seed: 16 Nucleotides

- Return: 2, TRIMA:0:31

CASE1B: CACATCATCGCTAGCTATCGATCGATCGATGCTATGCAAGATCGGAAGAGCT

||||||||------

AGATCGGAAGAGCT

- Seed: 8 Nucleotides

- Return: 2, TRIMA:0:37

CASE1C: CACATCATCGCTAGCTATCGATCGATCGATGCTATGCACGAAGATCGGAAGA

||||||||---

AGATCGGAAGA

- Seed: 8 Nucleotides

- Return: 1, reason: Match length < 12

CASE2A: CATACATCACGAGCTAGCTAGAGATCGGAAGAGCTCGTATGCCCAGCATCGA

||||||||||||||||------

AGATCGGAAGAGCTCGTATGCC

- Seed: 16 Nucleotides

- Return: 0, reason: remaining read too short.

CASE2B: CCACAGTACAATACATCACGAGCTAGCTAGAGATCGGAAGAGCTCGTATGCC

||||||||||||||||||||||

AGATCGGAAGAGCTCGTATGCC

- Seed: 16 Nucleotides

- Return: 2, TRIMA:0:28

CASE3A: TATGCCGTCTTCTGCTTGCAGTGCATGCTGATGCATGCTGCATGCTAGCTGC

||||||||||||||||--

TATGCCGTCTTCTGCTTG

- Seed: 16 Nucleotides

- Return: 0, reason: remaining read too short

CASE3B: CGTCTTCTGCTTGCCGATCGATGCTAGCTACGATCGTCGAGCTAGCTACGTG

||||||||-----

CGTCTTCTGCTTG

- Seed: 8 Nucleotides

- Return: 0, reason: remaining read too short

CASE3C: TCTTCTGCTTGCCGATCGATGCTAGCTACGATCGTCGAGCTAGCTACGTGCG

||||||||---

TCTTCTGCTTG

- Seed: 8 Nucleotides

- Return: 1, reason: Match length < 12

## ◆ is\_read\_inBloom()

|  |  |  |  |
| --- | --- | --- | --- |
| bool is\_read\_inBloom | ( | Bfilter \* | *ptr\_bf*, |
|  |  | Fq\_read \* | *seq*, |
|  |  | Bfkmer \* | *ptr\_bfkmer* |
|  | ) |  |  |

checks if a read is in Bloom filter. It computes the score for the read and returns true if it exceeds the user selected threshold. Returns false othersise.

Parameters
:   |  |  |
    | --- | --- |
    | ptr\_bf | pointer to Bfilter |
    | seq | fastq read |
    | ptr\_bfkmer | pointer to Procs\_kmer structure (will store global) |

Returns
:   true if read was found, false otherwise

## ◆ is\_read\_inTree()

|  |  |  |  |
| --- | --- | --- | --- |
| bool is\_read\_inTree | ( | Tree \* | *tree\_ptr*, |
|  |  | Fq\_read \* | *seq* |
|  | ) |  |  |

check if Lread is contained in tree. It computes the score for the read and its reverse complement; if one ot them exceeds the user selected threshold, it returns true. Otherwise, it returns false.

Parameters
:   |  |  |
    | --- | --- |
    | tree\_ptr | pointer to Tree structure |
    | seq | fastq read |

Returns
:   true if read was found, false otherwise

## ◆ Nfree\_Lmer()

|  |  |  |  |
| --- | --- | --- | --- |
| static int Nfree\_Lmer | ( | Fq\_read \* | *seq*, |
|  |  | int | *minL* |
|  | ) |  |  |

static

Finds the largest Nfree sub-seq and keeps it if larger than minL.

Parameters
:   |  |  |
    | --- | --- |
    | seq | fastq read |
    | minL | minimum accepted trimmed length |

Returns
:   0 if not used, 1 if accepted as is, 2 if accepted and trimmed

## ◆ no\_lowQ()

|  |  |  |  |
| --- | --- | --- | --- |
| static int no\_lowQ | ( | Fq\_read \* | *seq*, |
|  |  | int | *minQ* |
|  | ) |  |  |

static

checks if a sequence contains lowQ nucleotides

Parameters
:   |  |  |
    | --- | --- |
    | seq | fastq read |
    | minQ | minimum accepted quality value |

Returns
:   0 if seq contains lowQ nucleotides, 1 otherwise

## ◆ no\_N()

|  |  |  |  |  |  |
| --- | --- | --- | --- | --- | --- |
| static int no\_N | ( | Fq\_read \* | *seq* | ) |  |

static

checks if a sequence contains any non standard base callings (N's)

Returns
:   0 if no N's found, 1 if N's found

This function checks if any of the base callings in a given fastq read is different from A, C, G, T. Basically, any char different from the former ones is classified as N.

## ◆ Ntrim\_ends()

|  |  |  |  |
| --- | --- | --- | --- |
| static int Ntrim\_ends | ( | Fq\_read \* | *seq*, |
|  |  | int | *minL* |
|  | ) |  |  |

static

trims a read if N's are at the ends and the remaining sub-seq >= minL

Parameters
:   |  |  |
    | --- | --- |
    | seq | fastq read |
    | minL | minimum accepted trimmed length |

Returns
:   0 if not used, 1 no N's found, 2 if accepted and trimmed

## ◆ Qtrim\_ends()

|  |  |  |  |
| --- | --- | --- | --- |
| static int Qtrim\_ends | ( | Fq\_read \* | *seq*, |
|  |  | int | *minQ*, |
|  |  | int | *minL* |
|  | ) |  |  |

static

trims a read if lowQs are at the ends and remaining sub-seq >= minL

Parameters
:   |  |  |
    | --- | --- |
    | seq | fastq read |
    | minQ | minimum accepted quality value |
    | minL | minimum accepted trimmed length |

Returns
:   0 if not used, 1 if accepted as is, 2 if accepted and trimmed

## ◆ Qtrim\_frac()

|  |  |  |  |
| --- | --- | --- | --- |
| static int Qtrim\_frac | ( | Fq\_read \* | *seq*, |
|  |  | int | *minQ*, |
|  |  | int | *nlowQ* |
|  | ) |  |  |

static

accepts the sequence as is if there are less than nlowQ

Parameters
:   |  |  |
    | --- | --- |
    | seq | fastq read |
    | minQ | minimum accepted quality value |
    | nlowQ | threshold on lowQ nucleotides (>= NOT allowed) |

Returns
:   0 if not used, 1 if accepted as is

## ◆ Qtrim\_global()

|  |  |  |  |
| --- | --- | --- | --- |
| int Qtrim\_global | ( | Fq\_read \* | *seq*, |
|  |  | int | *left*, |
|  |  | int | *right*, |
|  |  | char | *type* |
|  | ) |  |  |

trims left from the left and right from the right

Parameters
:   |  |  |
    | --- | --- |
    | seq | fastq read |
    | left | number of nucleotides to be trimmed from the left |
    | right | number of nucleotides to be trimmed from the right |
    | type | char indicating the type of trimming (Q,A). |

Returns
:   2, since they are all accepted and trim

## ◆ trim\_adapter()

|  |  |  |  |
| --- | --- | --- | --- |
| int trim\_adapter | ( | Fq\_read \* | *seq*, |
|  |  | Ad\_seq \* | *adap\_list* |
|  | ) |  |  |

trims sequence based on presence of N nucleotides

if (adapter length < 16) -> search for seeds 8 nucleotides long else -> search for seeds 16 nucleotides long if (seed found) -> calculate score if score > threshold -> aligner found, trim / discard and exit. else -> search for seeds 8 nucleotides long

Parameters
:   |  |  |
    | --- | --- |
    | seq | pointer to **Fq\_read** |
    | adap\_list | array of **Ad\_seq** |

Returns
:   -1 error, 0 discarded, 1 accepted as is, 2 accepted and trimmed

Note
:   Global input parameters from par\_TF are also used

## ◆ trim\_sequenceN()

|  |  |  |  |  |  |
| --- | --- | --- | --- | --- | --- |
| int trim\_sequenceN | ( | Fq\_read \* | *seq* | ) |  |

trims sequence based on presence of N nucleotides

Parameters
:   |  |  |
    | --- | --- |
    | seq | fastq read |

Returns
:   -1 error, 0 discarded, 1 accepted as is, 2 accepted and trimmed

This function calls a different function depending on the method passed as input par\_TF.trimN:

- NO(0): accepts it as is, (1),
- ALL(1): accepts it as is if NO N's found (1), rejects it otherwise (0),
- ENDS(2): trims the ends and accepts it if it is longer than minL (2 if trimming, 1 if no trimming), rejects it otherwise (0),
- STRIP(3): finds the longest N-free subsequence and trims it if it is at least minL nucleotides long (2 if trimming, 1 if no N's are found), rejects it otherwise (0).

## ◆ trim\_sequenceQ()

|  |  |  |  |  |  |
| --- | --- | --- | --- | --- | --- |
| int trim\_sequenceQ | ( | Fq\_read \* | *seq* | ) |  |

trims sequence based on lowQ base callings

Parameters
:   |  |  |
    | --- | --- |
    | seq | fastq read |

Returns
:   -1 error, 0 discarded, 1 accepted as is, 2 accepted and trimmed

This function calls a different function depending on the method passed as input par\_TF.trimQ:

- NO(0): accepts is as is , (1),
- FRAC(1): accepts it if less than par\_TF.nlowQ are found (1), rejects it otherwise (0),
- ENDS(2): trims the ends and accepts it if it is longer than minL (2 if triming, 1 if no trimming), rejects it otherwise (0),
- ENDSFRAC(3): trims the ends and accepts if the remaining sequence is at least minL bases long and if it contains less than nlowQ lowQ nucleotides (2 if trimming, 1 if no trimming). Otherwise, it is rejected, (0).
- GLOBAL(4): it trims globally globleft nucleotides from the left and globright from the right, (returns 2).

## Variable Documentation

## ◆ Nencode

int Nencode

global variable. Encoding for N's(\004)

## ◆ par\_TF

Iparam\_trimFilter par\_TF

global variable: Input parameters trimFilter.

global variable: Input parameters of makeTree.


---

Generated on Mon Mar 19 2018 23:42:01 for FastqPuri by  

 1.8.14
